# The Influence of Price on Purchase Intentions: Comparative Study between Cognitive, Sensory, and Neurophysiological Experiments

**DOI:** 10.3390/bs11020016

**Published:** 2021-01-25

**Authors:** Gabriel R. D. Levrini, Mirela Jeffman dos Santos

**Affiliations:** 1 Marketing Department, Escola Superior Propaganda e Marketing, São Paulo 04018-010, Brazil; 2 Programa de Pós-graduação Scricto Senso em Ensino de Ciências e Matemática, Department of Administrative Sciences, Universidade de Caxias do Sul–UCS, Caxias do Sul 95070-560, Brazil; mirelajs@gmail.com

**Keywords:** store brands, consumer perception, brand recognition, eye-tracker, facial reader

## Abstract

Price is considered one of the most important attributes in consumer’s choice. On the other hand, consumer’s knowledge about price tends to be imprecise. This study aims at providing new insights analyzing consumers’ perception of retail store brand (focused on Skin Care Products) comparing with two other skin care products, a premium, and a popular national brand. Second, to analyze the association price versus quality variables in the purchasing decision process. Third, to demonstrate the influence of both, unconscious and cognitive process during the purchase choice intention. Through Neuromarketing tools and protocols (quantitative and qualitative), the study exposes participants to a blind test of the three products and asks participants to talk about their sensory impressions like scent, feelings, and products texture. Using facial electromyography (EMG) and eye-tracker devices we measured consumers’ responses when we introduced price and brand name variables, by this way comparing unconscious and cognitive responses. The findings showed that an unconscious decision could be change when new variables were revealed. The study showed how conscious price variable was the major influence in their purchase intention.

## 1. Introduction

Traditionally, retail seeks differentiation by offering unique products and services or even new business formats. However, as suppliers could be the same for all retailers and business formats could be copied, it is necessary that retailers identify new ways of differentiating themselves from competitors. Consumers have impressions and images about the brands and these impressions have a great impact on the choice of stores for purchases and in the same way influence the purchasing behavior of these consumers [1] One of retail’s strategy differentiation is the development of store brands [2]. Literature asserts that Store Brands (SBs) could be understood as assign the store name to the products [3]. In addition, Calvo-Porral and Levy-Mangin (2016) [4] indicated that SBs are restricted to the store chain and linked to it in a single way, especially if the brand name is the same.

Several authors [5] pointed out that when customers have a favorable retailer’s brand image and trust it, they tend to transfer these attributes to SBs. In this way, SBs image can be understood as a set of features like store name, store services, prices, quality of merchandise, and knowledgeable salespeople [5,6] that meet consumers evaluation of the store, and, consequently, its SBs.

In the 1970s, SBs were considered low-priced products, with inferior quality, nonspecialized suppliers, and basic products priced at least 20% lower than the leading brands. Poor quality and price-based competition are characteristics of the first generation of store brands [7]. From the second SBs generation on, the concern in terms of quality levels began, although the focus remains on lower prices than those of competitors. Products started to have retail-related brands, and the price level remains below the category leaders (between 10 and 20% lower). With the third SBs generation, both the quality and the price level of SBs are close to the leading market national brand. In this phase, the strategy for adopting own brands was to follow the leaders (me-too). The competition starts to take place in terms of value, price, and quality. We are in the fourth SBs generation, where the retail SBs strategy is to offer value-added products, differentiated from competitors, developed with innovative technology. In this generation, SBs have the same (or even superior) image and quality as those of leading national brands but with a segmented offering [8].

The retail landscape has changed considerably in recent decades. Globally, it is estimated that totally SBs represent close to 15% of the total market. According to Nielsen Report [9], SBs growth has been higher in recent years almost globally, from 2015 to 2019 was 3.7% (year ago) and CAGR 2.5% (4 years), against 1.9% (year ago) and CAGR (4 years) 1% from branded products. For that reason, SBs are being called future “brand killers” or “Brand Disruption next phase” [10].

This research was delimited within SBs universe: we select to study Beauty and Personal Care category, in particular Skin Care products that had one of the highest growth rates under this scenario.

The justification for this choice is the past and projected growth of this category. The projected growth to Beauty and Personal Care products is 7.2% globally (CAGR 2019–2024) specifically in Asian and emerging markets, like Brazil where Beauty and Personal Care category increased 19.8% (from 2016 to 2018) and within these category, Skin Care products grew 101% (from 2016 to 2018) [11].

We are seeing an emerging rise of premium SBs products and it is reshaping retail strategies across the retail landscape. Instead of price, retailers’ current focus is on having SBs with quality equal to or better than national brands [12]. As perceived quality is generally seen as opposed to the price function [13], the evolution of SBs in quality products changed this relationship, varying perceptions of price and value.

This exploratory study proposes the following research questions:

- Do store brands (SBs) skin care products, meet the consumers expectations regarding price and quality association compared with premiums brands and popular brands?
- Will neurophysiological responses vary when compared with conscious actions in this context?

This research aims to analyze how prices influenced the purchase intentions comparing cognitive process, sensorial, and neurophysiological results.

First, we investigate sensorial perceptions and later the brand image perceptions an important process as consumers use images or previous experiences in their purchase process. We explore the concept of SBs price image and price consciousness, which considers the multidimensionality of perceptions about products [14].

## 2. Background

The gap between industry and retail is being reduced, either by new forms of consumption and new customers (millennials generation) or new technologies, new trends, and changes in markets dynamics. Loyalty had always been a treasured commodity for companies but now consumers have endless choice and omni channel access, their disloyalty, or brand switching, should worry manufacturers and retailers [15].

### 2.1. Price

Price is undoubtedly one of the most influential factors in the perceptions of products on the market. According to Beneke et al. (2015) [16], price is a real clue that consumers use in their purchasing decisions. The price variable is always present in the daily purchase and represents the value of the economic expenditure Zeithmal (1988) [17], refers as the sacrifice value) that must be given up by consumers to carry out a certain purchase transaction. Considering that the price represents “a sacrifice”, this variable has a contrary position to the purchase intention where generally the higher prices, the lower purchase possibilities. Although, in some cases, researchers like Nevin e Houston (1980) [18], Mitchell (2010) [19], and Kara, et al. (2009) [20] explained that consumers do not always see price in a negative way, which represents economic expenses, instead it is a complex variable on purchase decision. They suggest that price could be a signal of product quality and represents elegance and status. These price role perceptions, negative or positive, give rise to consumer’s price image [21].

The price image comprises a subjective and multidimensional concept, which involves emotional factors associated to products or services [14]. Thus, consumers perception about price is built through a complex process and does not necessarily reflect the real brand or product price. Therefore, the gap between the true price and the consumers’ perceptions about price can be large. The price can have a greater impact on purchase intentions when other extrinsic tips such as the brand-value (or product-value) or intrinsic tips related to the physical properties of the object are not clearly defined. Well-known brands, such as a strong extrinsic tip, have a symbolic level that positively affects their perception of value. Thus, when the brand name gives evidence of the company’s reputation, the consumer may prefer to use the brand to infer quality and value and consequently the purchase instead of the price [21].

There are two vital concepts when we think about the price image formation:

- Value consciousness: means the consumer evaluation in a purchase decision in which price paid is compared to the benefits received with the product or service [17].
- Price consciousness, which is the price perception for some consumers.

However, the term “price consciousness” has been broadly used to refer to consumer perception about price [17]; we use the term to refer to how much interest consumers have in saving money and, consequently, paying lower prices. Several researchers [22,23,24] presented the price awareness associated to the priority consumers attribute to pay low price, instead of other aspects like quality, design, style, and so on.

### 2.2. Prices, Brands, and Perceived-Value

The brand, then, is the tangible representation of a set of meanings, values, attributes, and experiences related to a product or service recognized and represented by a name, logo, and visual support language that defines its identity in a complete way [12]. According to Aaker (2002) [7], the brand must be a symbol, from which consumers perceived-value and particular attributes, which carries peculiar characteristics and makes the link between the individual and the brand.

The perceived-value means the balance between perceived benefits from the product compared to the sacrifices (financial, physical, time, and psychic) [17,20]. Such perception, then, involves an exchange between what the customer takes (e.g., level of quality, joy, well-being) and what he gives to buy, use, or consume the product (e.g., store services, product price, waiting lines, and other emotional feelings). This value evaluation can be understood as a continuum, which goes from a perception of the price as a simple monetary value paid to a complex process of choice [14].

The product value awareness influences the consumer’s purchase decision, since they use price information as a unit of measurement of value, although partial, to make their mental processing of choice [16].

In this context, the brand and the price level play a fundamental role in the quality evaluation and serve as heuristic hints. According to Vahie and Paswan (2006) [25], heuristic hints are generally used as a kind of mental shortcut to simplify the evaluation of an object or an event (experience), when individuals are not motivated to process information or when decisions are less important. In fact, research shows that when there are no other quality tips or inferences, the consumer has used the price and the brand to infer the quality of a product.

Previous studies which investigated the reasons store brands are successful (for example, [26,27] explained that similar quality of national brands and SBs is a decisive factor for consumers’ choice, especially if national brands had a higher price than SBs. In other words, it implies that the success of SBs depends not only on price but also on product quality [28]. The quality gap between national brands, premiums brands, and SBs has been decreasing (reflected by the growing of market share), the more consumers see value on a product, the greater the purchase intention [26].

### 2.3. Theory

The association between quality and price reflects the general belief of the consumer that the price level positively influences the level of product quality. While some authors argue that this association of quality and price varies between different categories [29], other authors [30,31], following Attribution Theory, argue that this association is product specific.

The Attribution Theory [31,32] explains that the low price of certain SBs products can lead consumers to attribute it to some dubious characteristic of the product, perceiving them as being of inferior quality. Thanasuta (2015) [33] argued that the more aware consumers are about price, the more engaged they are in buying SBs products. Santos et al. (2016) [34] pointed out that, considering the same product category, consumers are more likely to buy low price SB products instead of national brands offering higher prices. So, the combination of high quality and low-price tends to excite and attract consumers to buy SBs [6,21].

**Figure 1:**
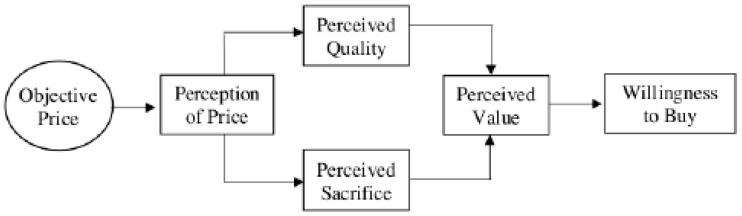
Price-value model proposed by Monroe and Krishman (1985) [35].

In their pioneering studies, Monroe and Krishnan (1985) [35] investigated the relationships among objective price, perception of price, perceived quality, perceived sacrifice, perceived-value, and willingness to pay. They proposed that perception of price rather than objective price positively affects consumers’ perceived quality and perceived sacrifice through the mediator of price perception (Figure 1).

However, Kwon (1990) [26] studied the influence of buying experience at the store on consumers’ perception of price, quality, and value. The results showed that store-shopping experience has a greater impact in consumers’ value perceptions of a retail store when compared to price or quality perceptions. Their findings confirmed the existence of nonproduct-related intrinsic attributes (e.g., consumers’ perceived store shopping experience), which may affect perceived-value (e.g., consumers’ perceptions of store value).

### 2.4. Consumers’ Perceptions of Price

Olson and Jacoby (1974) [36] and Jacoby, Olson and Haddock (1971) [37], in their first studies, reported that external stimuli do not directly affect behavior exercising only indirect effects. Stimuli must first be perceived and then decoded and interpreted before affecting behavior and decision-making. The perceived discount is defined as the coded discount through a subjective interpretation process that assigns meaning to the objective price or be the discounted price [4,36].

Following Jacoby and Olson (1977) [38] and Jacoby, Olson and Haddock (1971) [37] definitions of objective prices (which means the real price of a good) and perceived price (that is, the price consumers encode in their minds), [17] defined the perceived price, as the sacrifice to obtain a product. By this logic, the perceived price of consumers will be affected by the perceived discounts and in the same way by the perceived quality and the price image influenced by the brands power. The concept of perceived sacrifice, instead of perceived price, directly enters the process of evaluating consumers because the perceived-value is conceptualized as the compensation between perceived quality and perceived sacrifice [17,39]. Lindquist (1974) [40] argues that price image perceptions are influenced by the retailer’s store image and thus the store’s image would affect SBs. Some authors, however, do not agree with this explanation, arguing that price and store image are different concepts [14].

The store’s price image is defined as “a global representation of the relative price level” [17,39]. In this way, SBs price-image can be associated to costume price practiced by the retailer, according to its pricing strategies and positioning in the market. However, some authors identified many dimensions to the concept—like Zielke (2010) [14] and Lichtensteinetal (1993) [22]—considering that the price occupies a salient place in the consumer buying behavior process, especially for SBs.

In this regard, SBs price characteristics (for example, price awareness and value awareness) have been a discussion target of several researchers. SBs’ price image become a reference to consumers when purchasing a SB product. Retailers usually offer many types of SB products, such as luxury SBs, average SBs, economic SBs, and so on, varying quality and price [41,42].

We can infer that relying on the price image is more convenient for consumers than on any other price-related construct. Preceding studies showed that the SB image is a central element in development of store’s image [16,25,43]. Therefore, based on this discussion, we expect consumers to be influenced by the SB’s price image in their SB purchase decision making, since customer perceived-value means the balance between benefits received by the customer and costs paid by customer [42,44].

## 3. Material and Methods

The experimental sample was composed by three brands: store brand (S2), a premium brand (S3), and a national brand (S1). This choice aimed to compare consumers’ perceptions among different levels of quality, price, attributes, and perceived-value. S2 skin care was chosen as the SB, which was developed by the retailer and sought similar sensory attributes of a premium brand (S3) mainly in texture and aroma. To be consistent, we used S3 skin care as our premium sample and S1 skin care as the national brand sample. S1 is a very popular national brand in Brazil. All the experiments were done in Porto Alegre city, Brazil between August and October 2019.

### 3.1. Research Design

We divided the field research into two experimental stages.

The first stage was conducted by an exploratory qualitative research, using think-aloud protocols. At this time, participants were invited to try three skincare samples (Store Brand, National Brand and Premium Brand) in a blind test. During the test, participants were asked to say whatever comes into their mind as they complete their task. A total of 80 participants of our target group (women between 25 and 40 years old) participated in the experiment. Participants, all women, were recruited in their homes, as they all live in two populous condominiums in the same city and the neighborhood all middle-class social demographic segment. We invited them to participate and there was no payment; in fact, they enjoyed testing skin care products. We explained to the participants a general view of the experiment but not details in order not to jeopardize the experiment. 

At the end of the final session (5th), we invited them to a closing cocktail to celebrate the end of the experiment that took place when we reached our goal of 80 participants for establishing us in the design of the experiment.

The blind test was done individually in 5 sessions, in a control environment. During the individual experiment, we interviewed all the sample group recording their experience, utilizing the Talking Aloud protocols. Participants explained their experience and sensorial feelings.

Seeing that consumer purchase decision process is grounded by quality and price perceived, and that perceived-value is understood as the evaluation a consumer does about the benefits received and the costs paid for the product or service [17], at the end of each stage a basic question was asked: which product would you buy? 

The second stage was conducted with neurophysiological devices. The sample group were exposed to three products (premium, store brand, and national). We used eye tracker and facial electromyography (EMG) devices aiming to measure consumers’ emotional responses and to compare perceptions. While participants were looking at products labels, their eyes movements were registered at 60 Hz through the eye tracker equipment, which was integrated with the screen on which products were presented. The duration of each trial participant took on average 2 min as the three slides changed automatically every 20 s. Participants sat on a chair, which was at 65 cm from the screen and received the instruction to move as little as possible. Before starting each task, participants followed calibration procedure of Tobii Studio Professional version.

Finally, results included quantitative data, experimental setup, physiological and behavioral data collection, and statistical procedures.

### 3.2. Materials

For the experiment application, we used three skin care products that we maintain the anonymity of the brands: sample 1 (S1) a national brand, medium quality, low price, Sample 2 (S2) SBs of a large drugstore, with good quality, medium price, and sample 3 (S3) a premium brand with high quality and higher price among the samples.

For neurophysiological measurements, a biometric Imotions Platform was used, which has eye-tracking equipment Tobii 4.0 60 Hz, which monitors and describes the ocular movement of people, indicating which stimuli attract the most attention of an individual and providing the path that the participant’s eyes take when facing these stimuli, being therefore ideal for this study Eye tracking equipment is largely used in social science researches, because it offers data about individuals’ eyes fixation and visualization, which let researchers deeply analyze people’s behavior [45,46].

The *Tobii* device uses infrared light to illuminate the eyes of the study participants; the infrared light hits the user’s eyes and several sensors on the monitor capture them, allowing the software to interpolate the positioning of the eyes [47]. According to the authors, *Tobii* allows identifying with excellent accuracy where users are looking. The monitor is very similar to that of a common computer not necessarily being any type of additional apparatus coupled to the heads of the participants.

The facial EMG is an exact evaluation of facial muscle activity. This technique allowed analyzing spontaneous facial expressions that are deep emotions indicators.

**Figure 2:**
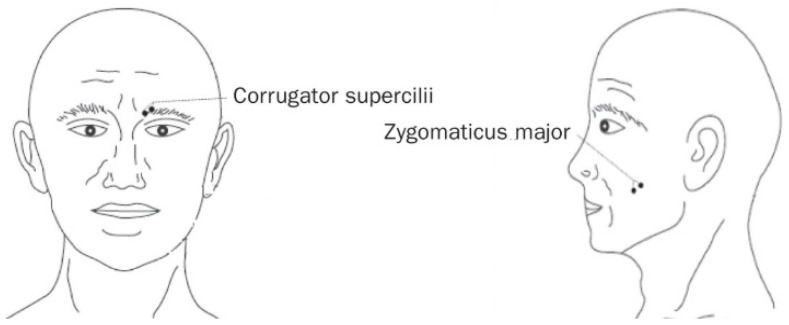
Placement of the electrodes. Source: Cacioppo, et al. (1999) [48].

To operationalize this procedure, we fixed sensors over defined individuals’ facial muscle, as shown in Figure 2, and observed muscle contraction through electrical current. This technique was already used to test individuals’ perception of television advertisements [48], radio advertisements [49], computer interfaces, new products [50], and internet [51]. Sensory inputs, such as those from pictures, slides, or videos, can be conducted straight to the amygdala (within limbic system) and to neocortex areas, where thought actions occur. According to previous research in neuromarketing area, emotional reactions are associated to unconscious brain zone when exposed to stimulus like advertisements or products pictures or brands films [52].

For current experiment, we considered two facial muscles: smile muscle—whose technical name is zygomatic major, representing a pleasant emotion—and frown—whose technical name is corrugator supercilii, representing an unpleasant emotion. Following Peacock, Purvis, and Hazlett (2011) [53] research, the activation was measured individually, because they are an evaluative indicative process, which had independent motivators of consumer behavior [54]. Positive activations influenced positive feelings, while Negative activation may expose negative emotion of viewer experiences.

Each participant sat in front of the equipment (Acer notebook with 32 Gg RAM, 2TB HD). At this moment, aspects such as distance between participant and monitor, height of the chair, and positioning of the person and environmental control variables was verified. 

**Figure 3:**
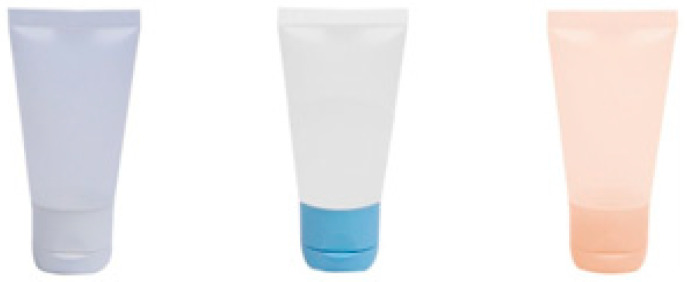
Slide A—Blind test transparent tubes. Source: the authors (2020).

**Figure 4:**
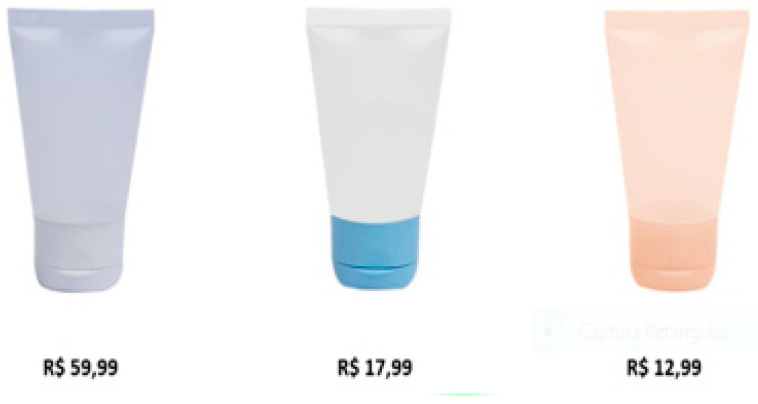
Slide B—Transparent tubes with prices. Source: the authors (2020).

**Figure 5:**
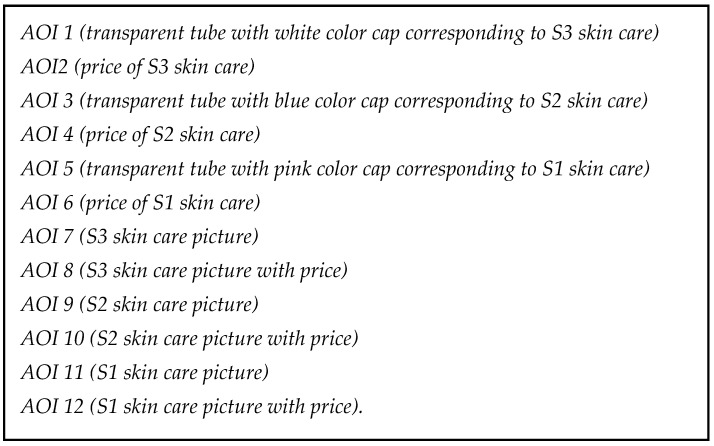
Areas of interest (AOIs) definitions. Source: the authors (2020).

The visual stimuli chosen was a set of three slides (A, B, C) that was exposed for 20 s each. Slide A (Figure 3) showed three transparent tubes containing the creams, only with different caps colors. Slide B (Figure 4) was the same three previous tubes, however, at this moment revealing market common prices (but without identifying the brand names); here, we seek to verify the physiological reactions when revealing the variable price. On slide C (Figure 5), we presented the three products with their respective attributes and prices, seeking insights about the perceived risk in relation to SBs.

At the end, we showed the last slide (C) where we introduced our coding (attributes specification) as S1 (pink cap), S2 (blue cap), S3 (white cap) (attributes) giving to the participants all the information about the products (excluding the brand name)

## 4. Results

### 4.1. First Stage Blind Test: Think Aloud Protocols

On first stage, basically, we explored only sensorial responses like smell, texture, and color of anonymous products (transparent tubes). In the blind test, we presented the three transparent tubes and invited the participants to try the creams, feel its texture and aroma, and say whatever comes into their minds during the experience. This procedure is named “Think-aloud” protocols and it involves asking people to use some product and tell about their experience, including feelings, sensations, impressions, and so on. Participants’ speeches were recorded for further analysis. This method is recommended because of the relaxed atmosphere it provides, which allows people to talk openly and informally about the products, being more sincere and faithful [55].

At the end, we asked the 80 participants which was the selected product and buying option. Twenty-five participants chose S2, 40 selected S3 and 15 elected S1, as summarized below (Box 1).

Box 1 **Purchase intention.**

**S2 (25/80)  S3 (40/80)  S1 (15/80)**

### 4.2. Second Stage: Neurophysiological Experiment

After the blind test, we submitted individually the participants to biometric devices. We presented the Slide A (they already known the transparent tubes from the blind test), and later we showed B and C slides, using eye-tracker and facial reader to understand their responses. Eye tracking’s objective is to identify areas of interest (AOI) and to measure for how long people fix their attention on specific areas. In this research, this methodology was used to analyze how people pay attention to prices and brand names. Eye-tracking devices help the researcher to determine what objects or parts of objects are being perceived and how they influence evaluation [56]. In our study, we defined specific detection parameters:

- Time to First Fixation (TTFF): It showed which areas were firstly looked by the participants and for how long the eyes were fixed on each AOI.
- Fixation: It identified people’s interests on slide or environment.
- Refixations: It measured how many times the participant looked at the same AOI. It is an important piece of data because it allows one to identify which areas call more attention from the participants (for better or worse) [57].
- Area of Interest (AOI)*:* Consists in dividing the display stimulus in selected areas and analyzing data for the specific regions. In this study, AOIs were defined as shown in Figure 5.

In slide B price figures were shown to analyze possible price versus quality (influence from the blind test) (AOIs 2, 4, and 6). We aimed in Table 1, Table 2 and Table 3 to measure mainly price effect for S3, S2, and S1 as we showed only the transparent tubes and prices as the following:

We found significant difference in almost all AOIs where prices were displayed, in fixation and refixation. In other words, it was no surprise that the premium brand, S3, was selected by most of participants, when it was anonymously presented in the blind test; however, when prices were revealed, participants purchase intention changed. When the price was showed, 19 participants chose S2, 16 selected S3, and 45 elected S1, as summarized below (Box 2).

Box 2 **Purchase Intention (with Price).**

**S2 (19/80)  S3 (16/80)  S1 (45/80)**

In Table 4, Table 5 and Table 6 we showed ANOVA test for Slide C when we revealed our coding (attributes) and prices.

We found significant difference between AOIs in almost all parameters measured. When coding was revealed, we expect some attributes’ influence reflecting in decision process and perceived-value. However, only four participants changed in favor of S3 and S1 national brand lost 14 participants. S2 increased the purchase intention in 10 participants, as summarized below (Box 3).

Box 3 **Purchase intention (with attributes).**

**S2 (29/80)  S3 (20/80)  S1 (31/80)**

### 4.3. EMG Results

Facial EMG data were analyzed by SPSS 20.0 software. Frown muscle registered more activation on Slide B (*M* = 345.21%), which reveled products’ prices. In sequence, Slide C (*M* = 268.01%), which showed products’ prices and attributes. The last one was the Slide A (*M* = 98.32%), which presented the transparent tubes already known for the participants. The differences among three slides were significative, in terms of their EMG mean, *F*(2, 64) = 14.26, *p* < 0.001, *n*_2_ = 0.31.

Post hoc tests using the Bonferroni correction, demonstrated that EMG mean responses to Slide B were significantly higher, than those for Slide C, which in turn, were significantly greater than for Slide A. Through ANOVA test, we found significant difference for the negative emotion measure between Slide A (without prices) and Slide B (with prices) (*F*(4, 10) = 2.98, *p* = 0.05) and also between Slide A and Slide C (*F*(1, 32) = 4.76, *p* = 0.03).

We did not find significant differences of positive emotion between Slide B (prices) and Slide C (attributes) (*F*(1, 31) = 2.23, *p* = 0.13), neither for Slide A nor C (*F*(1, 29) = 2.01, *p* = 0.18). Price variable reveled higher negative impression than attributes. These findings suggest, as we expected, that Slide B, showing prices, evoked more unpleasant emotions, followed by Slide C and Slide A.

## 5. Conclusions

In recent years, retail store brands (SBs) sales have grown faster than traditional brands, and in some cases have become market leaders (some later called “the future brand killers”). As an example, and focus of our research, we have Beauty and Personal Care category that remains one of the fastest growing categories. The rapid evolution of SBs happened mainly because retailers focused on product quality, without giving up the lower price characteristics of SBs. To reinforce this paradigm, in our research, we used a retail SB (S2 skin care) that was in its development, inspired in a premium brand (S3 skin care) both compared to a national brand (S1 skin care).

This exploratory study aimed to analyze how prices influenced the purchase intentions comparing cognitive process, sensorial, and neurophysiological results.

Our research had two stages: the first was a blind test (looking for perceptions and sensorial feelings) and the second stage used neurophysiological tools and cognitive responses.

This exploratory study proposes the following research questions:

- Do store brands (SBs) skin care products meet the consumers expectations regarding price and quality association compared with premium brands and national brands?

Consumer perception is an interaction of the characteristics (attributes) inherent to the product, the socioeconomic conditions, and physiological and cognitive influences. When the consumer is in the market, the purchase decision is influenced by the brand (or attributes) and it influences the evaluation response sensory. Prior study focused on the effect of the brand, by the price, product appearance, and sociocultural or general preference that could affect the sensory evaluation [58].

In the experimental blind test procedure, consumers hedonically tested the products and were invited to express their purchase intention. The consumers’ hedonic evaluation changed significantly from the first slide A (Blind test without revealing brand names) and the second slide B (Blind plus price). First, premium brand S3 was selected by 50% of participants, followed by the good quality S2 (31.25%) and national brand S1 (18.75%). This result is coherent with products quality. Sample S3 is considered the best skincare cream from the three samples presented. However, S1 is the simplest product, while S2 is a good quality product. So, the blind test revealed an expected consumers perception.

After revealing prices, in Slide B, the key question “which product would you buy?” was asked again and participants changed their option and selected national brand S1 (56%), they showed an increase option towards S2 (24%) and a decrease option for S3 (20%). At this point, we can see price influence over the purchase decision overlapping the previous sensorial selection. These results are aligned with Stefani, Romano, and Cavicchi (2006)’s previous research [59]. Furthermore, this result showed that participants, although preferred S3, are not willing to pay for it. In addition, their perceptions about the three creams on first stage were favorable, that is, participants enjoyed the three products and they chose the cheapest one.

These results showed that the disclosure of the attributes allowed participants to analyze the quality and the relevance of the products quickly and briefly [58]. When Slide C was showed and, again, we asked the key question, purchase option again slightly changed but at least showing that there exists an attributes’ influence and consciousness on consumer reply. When attributes are acknowledged, it generates an influence on the sensory acceptance of the product, that is, the consumer increases or decreases its valuation in relation to the knowledge or price positioning in the market. The final selection, after all the information revealed, showed the following purchase option: S1 decreased to 38.75%, S2 increased to 36.25% and S3 also increased to 25%. The results showed that consumers have expectations for certain attributes and in them generates different effects. In addition, this result suggests that consumers accept paying more for a skin care product since they know their complete attributes.

Consumers perceive high-quality products, generally founded as more expensive than medium or low-quality ones [60]. In our experiment it was possible to see purchase intention change towards a cheaper product (and against the blind test sensory choices) when prices were revealed, but later, when all the information was given, that is, the sample could be associated with price and product attributes, the purchase options changed again. Product price, however, had the major factor influencing in selecting the purchase intention. This distribution shows different consumers profiles: who values quality and attributes, who values low price, and the ones who value a balance between quality and price.

- Will neurophysiological responses vary when compared with conscious actions in this context?

In our research, it was clear the differences between the sensorial responses and the cognitive actions (especially when prices are disclosed). Sensorial responses suggested consumers’ preference for premium brand, which indicated that smell, texture, and color of this product were considered better than the others. In this way, participants had better sensations when they tried S3 skin care cream. However, when prices were reveled, participants made their purchase decision in a rational way and had cognitive actions, that way many of them changed their minds. In this way, neurophysiological responses were different from conscious actions.

### 5.1. Research Contributions

Our research has important academic contributions for consumer behavior studies mainly regarding the conscious impact of the price factor in the purchase intention process applied to skin care products. We take the opportunity to analyze three different products with a deep interest in retail SBs, due to its emerging market position.

This exploratory study proposes to respond to the following research questions:

- Do store brand (SB) skin care products meet the consumers’ expectations regarding price and quality association compared with premiums brands and popular brands? In general terms 36% of the participants chose the store brand, comparatively only the S3 (low price low quality) had a slightly higher level of choice of 38% which was a significant difference. This suggests that in terms of quality, participants were satisfied with the store brand; however, the price factor still had greater influence, possibly due to the current pandemic moment with high unemployment rates and financial difficulties of the population.
- Will neurophysiological responses vary when compared with conscious actions in this context? During the blind test and experiment first stage, the sensory influence was total, S2 (premium product) had a 50% choice, obviously the participants assimilated the best texture and aroma of the product. However, in the final stage when the price and attributes of all products were revealed, conscious choice had only 25% of choices, which was a significant difference *F*(183, 223) *p* < 0.001. That is, for the participants in their rational assessment in terms of the relationship between price and quality, the quality of the premium product was only recognized and valued by 25% of the participants in the experiment.

Our research in practical terms goes deeper into the concepts of price image, price consciousness, and price perception. The perceived-value represents the consumer evaluation of the benefits from the product compared to the sacrifices, which gives base of price perception. It was no surprise that the premium brand S3 was selected by 50% of participants, when it was anonymously presented in the blind test due to its intrinsic attributes (texture and aroma). When prices were revealed, participants’ purchase intention changed. This result suggests that people interviewed do not see value in a product whose price is double another one, which they also enjoy, although they preferred it in their initial choice. This change clearly reflects the concept of price consciousness. However, when all variables were revealed, a different understanding of price consciousness with the association of real price versus perceived quality and attributes was observed. Well-known attributes have strong extrinsic tip, a symbolic level that positively affects their perception of value.

The good quality S2 also reflects these changes. When prices were revealed (Slide B) its choice decreased; however, when attributes were revealed, its price versus quality association caused an increase in its choice. From neurophysiological experiment, it was clear that price variable reveled higher negative impression than attributes.

We understand that our finding could be interesting for retail managers.

In methodological terms, a differentiated way of capturing consumers’ perceptions was utilized, analyzing sensory responses, unconscious responses, and conscious responses applied at the same time. In practice, we saw in this experimental research how different variables could affect consumer decision in consumer products.

### 5.2. Limitations and Future Research Directions

We understand this research has some limitations. Generally, in accordance with Orquin, Ashby, and Clarke (2016) [61], eye-tracking method considers fixations to identify visual attention, focused on an individual’s foveal vision. However, this tool despises peripheral vision, which limits the analyses of real attention paid on each zone of the image. In other words, the fact that they are not fixations in AOIs does not mean that they are not aware that it was there. Still, it may be that some information presented in their visual field was not focused on it. Although participants had fixed their eyes on specific AOI, we cannot affirm that it was all they had observed. We considered a random sample of 80 individuals only, so it is a small sample for generalization.

That said, we suggest for future studies to continue this investigation to cover our limitations. It would be important to expand participants to other regions and countries. Sample enlargement will allow a deeper investigation, building hypothesis and testing them by structural equation modeling.

## Figures and Tables

**Table 1:** AOI 2 ANOVA test S3 Price.

		χ 2	σ	df	F	Sig.
TTFF						
	Within AOI	14,775	1.934	1	3.872	0.13
	Between AOIs	390,625	0.878	79	100,875	0.01 **
FIXATION						
	Within AOI	133,112	0.899	1	1,767	0.08
	Between AOIs	462,776	0.799	79	122,443	0.05 *
RE-FIXATION						
	Within AOI	189,332	1.221	1	3,224	0.05 *
	Between AOIs	473,223	2.331	79	179,998	0.01 **

** significative at 99%, * significative at 95%.

**Table 2:** AOI 4 ANOVA test S2 Price.

		χ 2	σ	df	F	Sig.
TTFF						
	Within AOI	13,891	0.891	1	2	0.11
	Between AOIs	398,222	1.12	79	98,221	0.05 *
FIXATION						
	Within AOI	113,987	0.781	1	4332	0.01 **
	Between AOIs	477,654	0.886	79	154,222	0.01 **
RE-FIXATION						
	Within AOI	197,334	0.88	1	5233	0.01 **
	Between AOIs	489,011	0.79	79	165,112	0.01 **

** significative at 99%, * significative at 95%.

**Table 3:** AOI 6 ANOVA test S1 Price.

		χ 2	σ	df	F	Sig.
TTFF						
	Within AOI	10,322	0.81	1	2	0.16
	Between AOIs	287,009	0.98	79	121,223	0.05 *
FIXATION						
	Within AOI	100,233	1.12	1	3887	0.05 *
	Between AOIs	336,887	1.05	79	158,998	0.01 **
RE-FIXATION						
	Within AOI	119,02	0.776	1	4112	0.01 **
	Between AOIs	244,898	0.887	79	182,998	0.01 **

** significative at 99%, * significative at 95%. Source: the authors (2020).

**Table 4:** AOI 8 ANOVA test S3 Price.

		χ 2	σ	df	F	Sig.
TTFF						
	Within AOI	19,729	0.998	1	3	0.13
	Between AOIs	338,983	0.789	79	121,223	0.05 *
FIXATION						
	Within AOI	34,509	0.99	1	4334	0.05 *
	Between AOIs	499,221	1.03	79	182,443	0.01 **
RE-FIXATION						
	Within AOI	16,112	1.08	1	1223	0.12
	Between AOIs	334,224	1.34	79	168,999	0.01 **

** significative at 99%, * significative at 95%.

**Table 5:** AOI 10 ANOVA test S2 Price.

		χ 2	σ	df	F	Sig.
TTFF						
	Within AOI	98,781	1.02	1	1	0.15
	Between AOIs	135,788	0.98	79	118,333	0.05 *
FIXATION						
	Within AOI	128,881	0.94	1	3887	0.05 *
	Between AOIs	497,344	1.02	79	202,11	0.05 *
RE-FIXATION						
	Within AOI	212,371	0.96	1	3566	0.11
	Between AOIs	398,221	0.97	79	164,22	0.05 *

* significative at 95%.

**Table 6:** AOI 12 ANOVA test S1 Price.

		χ 2	σ	df	F	Sig.
TTFF						
	Within AOI	21,233	1.12	1	2	0.18
	Between AOIs	366,221	1.05	79	176,334	0.05 *
FIXATION						
	Within AOI	98,887	1.02	1	3455	0.05 *
	Between AOIs	287,478	0.95	79	188,322	0.01 **
RE-FIXATION						
	Within AOI	103,12	1.02	1	1443	0.17
	Between AOIs	221,223	0.99	79	3556	0.05 *

** significative at 99%, * significative at 95%. Source: the authors (2020).

## Data Availability

All the data presented in this paper is original, were not manipulated or inappropriately selected, enhanced, fabricated and the analysis tools or methods support all conclusions obtained.
